# A process for assessing the feasibility of a network meta-analysis: a case study of everolimus in combination with hormonal therapy versus chemotherapy for advanced breast cancer

**DOI:** 10.1186/1741-7015-12-93

**Published:** 2014-06-05

**Authors:** Shannon Cope, Jie Zhang, Stephen Saletan, Brielan Smiechowski, Jeroen P Jansen, Peter Schmid

**Affiliations:** 1 Mapi, 33 Bloor Street East, Suite 1300, Toronto, Ontario M4W 3H1, Canada; 2 Novartis Pharmaceuticals Corporation, One Health Plaza, BLDG 337, A10.4C, East Hanover, NJ 07936, USA; 3 Mapi, 180 Canal Street, Suite 503, Boston, MA 02114, USA; 4 Tufts University School of Medicine, 145 Harrison Ave, Boston, MA 02111, USA; 5 Barts Cancer Institute, Queen Mary University of London, Old Anatomy Building, Charterhouse Square, London EC1M6BQ, UK

**Keywords:** Advanced breast cancer, Everolimus, Chemotherapy, Network meta-analysis, Progression-free survival, Systematic literature review, Feasibility assessment

## Abstract

**Background:**

The aim of this study is to outline a general process for assessing the feasibility of performing a valid network meta-analysis (NMA) of randomized controlled trials (RCTs) to synthesize direct and indirect evidence for alternative treatments for a specific disease population.

**Methods:**

Several steps to assess the feasibility of an NMA are proposed based on existing recommendations. Next, a case study is used to illustrate this NMA feasibility assessment process in order to compare everolimus in combination with hormonal therapy to alternative chemotherapies in terms of progression-free survival for women with advanced breast cancer.

**Results:**

A general process for assessing the feasibility of an NMA is outlined that incorporates explicit steps to visualize the heterogeneity in terms of treatment and outcome characteristics (Part A) as well as the study and patient characteristics (Part B). Additionally, steps are performed to illustrate differences within and across different types of direct comparisons in terms of baseline risk (Part C) and observed treatment effects (Part D) since there is a risk that the treatment effect modifiers identified may not explain the observed heterogeneity or inconsistency in the results due to unexpected, unreported or unmeasured differences. Depending on the data available, alternative approaches are suggested: list assumptions, perform a meta-regression analysis, subgroup analysis, sensitivity analyses, or summarize why an NMA is not feasible.

**Conclusions:**

The process outlined to assess the feasibility of an NMA provides a stepwise framework that will help to ensure that the underlying assumptions are systematically explored and that the risks (and benefits) of pooling and indirectly comparing treatment effects from RCTs for a particular research question are transparent.

## Background

Network meta-analyses (NMA) are increasingly being performed to inform decision-making regarding the comparative efficacy and safety of alternative treatments [1]. In order to determine the comparative efficacy or safety of a new treatment using a NMA it is necessary to establish the relevant comparators. Generally, the indication for the new treatment and the way in which the new treatment is expected to be used in clinical practice will determine the comparators of interest. In some cases the comparators are explicitly defined by reimbursement agencies for a technology appraisal, which is the case in the United Kingdom where the National Institute for Health and Care Excellence (NICE) develops a final scope based on a stakeholder consultation process [2].

In order to inform decision-making it is necessary to assess whether it is feasible to perform a valid NMA to compare the new treatment with usual care based on the available randomized controlled trials (RCTs). As with any NMA, the validity of such analysis relies on whether there are systematic differences among the studies included in the network across treatment comparisons, especially patient or disease characteristics that are treatment effect modifiers [3-6]. Although there is guidance available regarding the underlying principles of an NMA, there is a need for a more structured process that incorporates both clinical and methodological expertise to assess the feasibility of performing a valid NMA [7]. The aim of this study is to outline a general process for assessing the feasibility of performing a valid NMA. A case study is used to illustrate the feasibility of performing an NMA to compare everolimus in combination with hormonal therapy to alternative chemotherapies in terms of progression-free survival (PFS) for women with advanced breast cancer (ABC).

The first section presents general steps for assessing the feasibility of a NMA. Next, the case study is presented in terms of the background, the identification and selection of trials, the method for the systematic review and analysis, and the results of the feasibility assessment and NMA. Readers are encouraged to use our application of these rules to our clinical example as a case study in applying our process to a possible research question and may envision ways to apply it to their own review.

## Methods

### Assessing feasibility of a network meta-analysis

In the absence of trials involving a direct comparison of interventions, an indirect comparison can provide valuable evidence for the relative treatment effects between competing interventions [5,8-14]. Even when the results of the direct evidence are conclusive, combining them with the results of indirect estimates in a mixed treatment comparison may yield more precise estimates as a greater evidence base is considered [8,10,12]. If the available evidence base consists of a network of interlinked multiple RCTs involving treatments compared directly or indirectly or both, it can be synthesized by means of NMA [4,8,11,13,14]. Since randomization of patients does not hold across trials in a network of RCTs, there might be differences across treatments that may compromise the validity of a NMA. It can be expected that there will always be some degree of variation in patient characteristics across studies. If these characteristics are effect modifiers of the relative treatment effects of interest then there will be heterogeneity in the evidence base. If there is an imbalance in relative treatment effect modifiers across comparisons, then the transitivity and consistency assumptions do not hold and some or all of the estimates of the NMA will be biased [5,9,11]. Therefore, it is important to assess whether there are differences in study and patient characteristics across comparisons that affect the summary measures of treatment effects (that is, odds ratio or hazard ratio) for the interventions of interest relative to an overall reference treatment. (Note: Differences in prognostic factors that are not also treatment effect modifiers do not impact the validity of the analysis).

**Figure 1:**
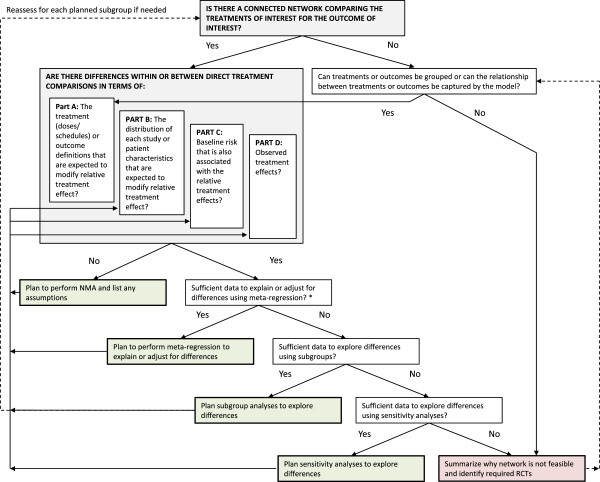
**Overview of process to assess the feasibility of performing a valid network meta-analysis.** *Planned meta-regressions in previous steps should be considered when assessing whether there is sufficient data for a meta-regression. It may be possible to perform separate meta-regressions per potential treatment effect modifier, although this should be clearly stated as a limitation.

A general process for assessing the feasibility of an NMA is outlined in Figure 1, which builds upon the existing recommendations regarding NMA [5,6,11]. Initially, steps to visualize the clinical heterogeneity in terms of treatment and outcome characteristics (Part A) as well as the study and patient characteristics (Part B) are proposed [7]. Next, steps are suggested to assess differences within and across the direct pairwise comparisons in terms of baseline risk (Part C) and observed treatment effects (Part D) since there is a risk that the treatment effect modifiers identified may not explain the observed heterogeneity or inconsistency in the results due to unexpected, unreported or unmeasured differences.

The proposed process for the feasibility assessment of an NMA is recommended to be specified in the systematic review protocol and statistical analysis plan. It is recommended to develop a parsimonious list of potential treatment effect modifiers for the study and patient characteristics based on prior knowledge before beginning the systematic review and feasibility assessment that will help guide Part B [6]. The process also acknowledges that some additional differences may be identified that act as treatment effect modifiers, which could be predefined in terms of a process to identify outliers. In some cases a modification to the statistical analysis plan may be required. For example, decisions regarding pooling different treatment doses or regimens (Part A) may be challenging to pre-specify in the statistical analysis plan without having identified the relevant evidence.

It is recommended that the decision to perform an NMA should be based primarily on clinical judgment of whether differences among studies may affect the comparisons of treatments or make some comparisons inappropriate [11]. However, an evaluation of baseline risk and heterogeneity (or inconsistency) in observed treatment effects in Part C and Part D may help identify analyses to adjust for differences [4,15]. Therefore, it is suggested to pre-specify the types of analyses that will be used to explore heterogeneity and/or inconsistency, which may include the use of a random effects model, unrelated means model [4], exclusion of specific studies that are outliers (using pre-defined criteria), node splitting [16], or the inclusion of inconsistency factors [17-19]. Ultimately, using this process will help to ensure that the risks (and benefits) of pooling and indirectly comparing treatment effects reported in RCTs for a particular research question are clearly documented [11].

An important step in the feasibility assessment involves making a judgment on whether or not there is sufficient data to explain or adjust for differences in potential treatment effect modifiers using a meta-regression. Although there is no clear threshold regarding the number of data points required to perform a meta-regression, Gagnier *et al*. refer to a general rule of thumb which suggests that there should be close to ten trials when working with summary or aggregate patient data (or ten individuals per variable, when working with pooled or individual patient data), but also caution that fewer studies may be associated with more heterogeneity, and more variables explored may be associated with a higher type 1 error rate [7]. Therefore, the number of data points should be considered against the number of parameters included in the meta-regression model. Generally, when these types of meta-regressions are based on aggregate study level data, it is assumed that the effect of the covariate is constant across the different treatments, which is recommended for most cases given the limited data available [20]. While accounting for study-level factors using these types of models is advised, the risk of ecological bias should be recognized when adjusting for differences in patient characteristics in the absence of individual patient level data [21,22].

### Case study

Everolimus is indicated for the treatment of hormone receptor-positive, human epidermal growth factor receptor type 2 negative (HER2-) ABC, in combination with exemestane (EXE), in postmenopausal women without symptomatic visceral disease after recurrence or progression following a non-steroidal aromatase inhibitor (NSAIs) [23].

The phase III RCT BOLERO-2 demonstrated that everolimus plus EXE more than doubled median PFS compared with placebo plus EXE while still maintaining quality of life in ABC patients (estrogen receptor positive (ER+) and HER2-) who recurred or progressed during or after NSAIs [24-27]. Additionally, the phase II TAMRAD trial demonstrated the efficacy of everolimus in combination with tamoxifen (TAM) in comparison to TAM alone [28].

**Figure 2:**
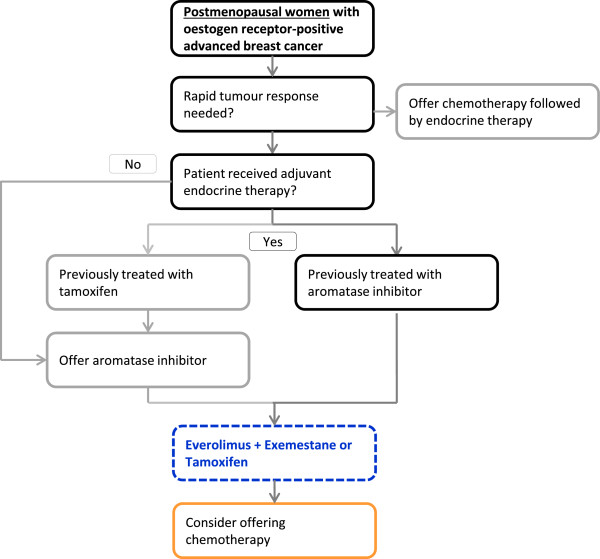
**Anticipated treatment pathway including everolimus for advanced breast cancer.** Adapted from NICE Pathways, Advanced Breast Cancer: endocrine therapy; Accessed July 22 2013 from: [32].

For women with ER+ ABC, guidelines recommend endocrine therapy as the preferred option even in the presence of visceral disease. If there is evidence of endocrine resistance or rapidly progressive disease requiring a fast response then chemotherapy is recommended [29]. Figure 2 illustrates the current treatment pathway for ER+ patients with ABC and also outlines the anticipated use of everolimus in the treatment sequence. Everolimus in combination with EXE offers a viable new line of therapy which can delay treatment with chemotherapy and has been described as ‘a step change in treatment’ [30]. Consequently the comparison of everolimus in combination with hormonal therapy versus chemotherapy is of interest and was defined as a comparator of interest by NICE [31].

#### Identification and selection of studies

A systematic literature search was performed in March 2013 to identify published RCTs evaluating the efficacy of treatment regimens for patients with postmenopausal ABC (stage III or IV) who were treated with everolimus, alternative hormonal therapies or alternative chemotherapies to facilitate an indirect comparison of everolimus versus chemotherapy. Medline, Medline In-Process, EMBASE and Cochrane databases were searched by using a predefined search strategy with terms relevant to ABC, RCTs and the comparisons of interest (see search strategy in Additional file 1).

Two reviewers independently evaluated each identified study against the following predetermined criteria:

Population: postmenopausal women with ABC (locally ABC (stage III) or metastatic breast cancer (stage IV)).

Interventions: everolimus (in combination with hormonal therapy) and chemotherapies including capecitabine, vinorelbine, paclitaxel, docetaxel, nab-paclitaxel, doxorubicin, epirubicin, pegylated liposomal doxorubicin and eribulin.

Comparisons Step 1: everolimus (in combination with hormonal therapy) versus hormonal therapy alone; EXE versus TAM; hormonal therapy versus chemotherapy.

Comparisons Step 2: chemotherapy identified in Step 1 versus any chemotherapies of interest; Comparison of alternative hormonal therapies identified in Step 1.

Outcomes: Kaplan Meier curves reporting PFS, time to progression (TTP) or time to failure (TTF).

Study design: phase II or III RCTs.

Although the target population for everolimus was based on the BOLERO-2 trial, which included only ER+ HER2- patients with prior NSAI treatments, the scope of the population was defined more broadly in order to capture all available evidence for trials comparing hormonal therapy to chemotherapy.

For each identified study that met the selection criteria, details were extracted on study design, study population characteristics and interventions. For all studies, the reported PFS Kaplan-Meier curves were digitized for each treatment arm (DigitizeIt v1.6.1). The data set was created on the basis of extracted progression proportions (including PFS, TTP or TTF), which were used to calculate the incident number of events for each interval and patients at risk at the beginning of that interval [33]. Generally, PFS is defined as the time elapsed between randomization and tumor progression or death from any cause, with censoring of patients who are lost to follow-up, where progression events include an increase in tumor size and/or the development of new tumors according to standardized criteria such as Response Evaluation Criteria in Solid Tumors. TTP is typically defined as the time elapsed between randomization and tumor progression, with censoring of patients who die or are lost to follow-up. Finally, TTF may be defined as the time from randomization until progression, relapse, or death from any cause, although this may vary. PFS, TTP and TTF are not always labelled or defined consistently in different trials [34].

#### Assessing the feasibility of a network meta-analysis for postmenopausal women with advanced breast cancer

The process outlined in Figure 1 was applied in order to assess whether a NMA was feasible to indirectly compare everolimus (in combination with hormonal therapy) to alternative chemotherapies in terms of PFS for postmenopausal women with ABC. The following potential treatment effect modifiers were identified *a priori* based on clinical expertise: hormone receptor status (HR-status), prior hormonal therapy, prior chemotherapy, visceral metastases, performance status and age. For parts A and B, network diagrams illustrating the structure of the network as well as differences in outcome definitions and potential treatment effect modifiers were developed. For parts C and D the baseline risk and heterogeneity in observed treatment effects were also illustrated to facilitate an assessment of the differences within and across direct treatment comparisons.

A Bayesian NMA was planned using the methodology introduced by Ouwens *et al*. and Jansen *et al*. to synthesize and indirectly compare the published PFS Kaplan-Meier curves for each treatment and RCT [33,35,36]. With this approach, the PFS of patients over time of the interventions compared in a trial is modeled with parametric survival functions and the difference in the shape and scale parameters of these functions between interventions is synthesized and indirectly compared across trials. The best fitting first order fractional polynomial model was selected [36] using the deviance information criteria (DIC) [37,38]. Additional details on these models and developing the datasets have been reported previously [33,35,36,39].

## Results

### Study identification

**Figure 3:**
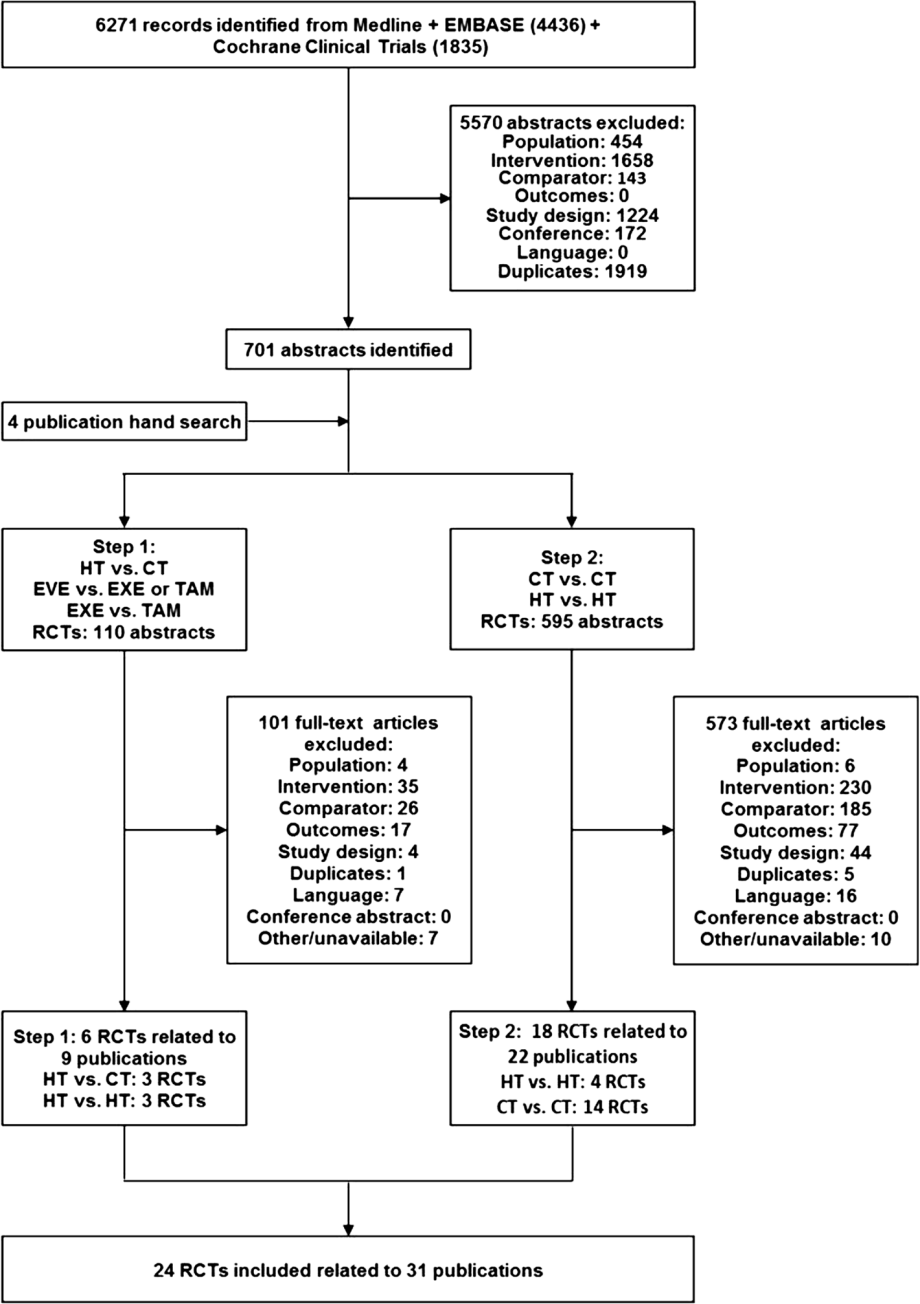
**Flow chart illustrating the study selection process for the systematic review.** CT, chemotherapy; EVE, everolimus; EXE, exemestane; HT, hormonal therapy; RCTs, randomized controlled trials; TAM, tamoxifen.

A stepwise process was used to select the relevant RCTs and to assess the feasibility of a NMA (Figure 3 presents the flow chart, while Additional file 2: Table S1 presents all included trials, their respective authors, years of publication and interventions) [24-28,40-64]. Based on the first step of the systematic literature review three RCTs were identified comparing hormonal therapies to chemotherapies (TAM versus cyclophosphamide + doxorubicin (CD), megestrol acetate (MA) versus cyclophosphamide + methotrexate + fluorouracil (CMF), and MA versus mitoxantrone (MZ) [40,43,64]). These RCTs were connected to three RCTs identified comparing everolimus + TAM to TAM, everolimus + EXE to EXE, and TAM to EXE [24,25,28,59-62]. Therefore, it was possible to develop a network of connected RCTs based on these six RCTs. However, this network, based on step 1, only allowed for an indirect comparison of everolimus (plus hormonal therapy) to the chemotherapies CD and CMF. Since these chemotherapy combinations were not of interest, the network was extended to include an additional 14 RCTs comparing alternative chemotherapies of interest [41,42,45-47,49,50,54,57-60,63],[65,66].

Additionally, four RCTs comparing alternative hormonal therapies in the network were included to strengthen the network (comparing MA to TAM or EXE) [43,48,51-53,55,56] based on the final step outlined in the systematic review protocol. Although the objective of this network was not to compare the efficacy of alternative hormonal therapies, these RCTs provided additional connections between trials evaluating everolimus and those treatments required to link to chemotherapies of interest. As was done for this case study, we would advise the utilization of the most ‘comprehensive’ evidence base in the initial feasibility assessment. However, for trials comparing treatments that are not directly of interest for the decision-problem it is important to consider that the additional value of evidence should be weighed against the risk of introducing additional heterogeneity (or inconsistency) to the network. Exploring the impact of a broader evidence base for the network was not the purpose of this study, although this is an important issue that has been considered by Cooper *et al*. [67].

### Is there a connected network comparing the treatments of interest for the outcome of interest?

The available RCTs formed a connected network of evidence in order to indirectly compare everolimus in combination with hormonal therapy to the relevant chemotherapies, which is illustrated in Additional file 3: Figure S1. However, the indirect comparison of everolimus (in combination with hormonal therapy) versus any of the chemotherapies of interest is mediated by at least four different treatment comparisons. The studies included between 48 to 769 patients per study, with five studies including fewer than 100 patients. Based on the number of relatively small RCTs available and the large ‘degree of separation’ in the network pathway connecting the treatments of interest, the estimates of the indirect treatment were expected to be very uncertain.

### Are there differences within or between direct treatment comparisons for the outcome of interest?

#### Part A - The treatment (doses/ schedules) or outcome definitions that are expected to modify relative treatment effect

Some differences in the treatment doses and/or schedules were identified, which are summarized in Additional file 4: Table S2. Since there were a limited number of RCTs included per treatment (ranging from one up to seven RCTs for doxorubicin), a meta-regression was not deemed to be feasible to adjust for differences in treatment doses. However, the RCTs that were most different in terms of treatment dose were planned to be excluded in a sensitivity analysis based on clinical expertise. In the ‘All Evidence’ base case analysis the different treatment doses were grouped together assuming that there would have been no differences in outcomes (beyond sampling error) between the different treatments within the group if the same population would have been treated. A comparison between the base case analysis and the sensitivity analysis excluding specific treatment doses may be possible to explore the impact of this assumption.

**Figure 4:**
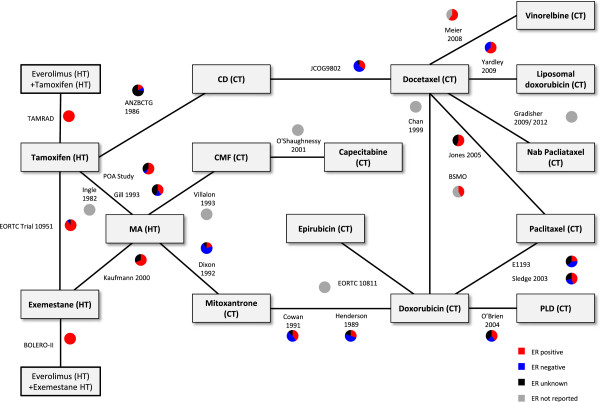
**Network of included RCTs for the base case PFS based on Kaplan Meier curves: hormone receptor status.** CD, cyclophosphamide + doxorubicin; CMF, cyclophosphamide + methotrexate + 5-fluorouracil; CT, chemotherapy; ER, estrogen receptor; HT, hormonal therapy; MA, megestrol acetate; PFS, progression-free survival; PLD, pegylated liposomal doxorubicin; RCT, randomized controlled trial*.*

Differences across the RCTs and treatment comparisons were also identified in terms of the outcome definition. Eleven RCTs reported TTP, seven RCTs reported PFS and six RCTs reported TTF (Figure 4). The network included 24 trials evaluating two treatment arms of interest in terms of 17 different treatments. Therefore, the simplest arm-based (fixed effect) model assuming a constant hazard ratio to synthesize the network of studies would include 40 parameters (24 baseline effects for each study and (17–1 = 16) treatment effects), whereas an analysis based on reported hazard ratios would provide only 24 data points. A meta-regression to adjust for differences in the PFS definitions was planned but may not be feasible given the limited amount of data; therefore, a sensitivity analysis was planned to exclude RCTs within the network evaluating TTF [40,44,48,50,63,68], which may differ the most from TTP and PFS.

#### Part B – The distribution of study or patient characteristics that are expected to modify relative treatment effects (defined a priori)

RCTs in the network were generally multicenter open-label trials evaluating women with either advanced or recurrent breast cancer and the study design was considered to be broadly comparable despite some single center trials [43,64]. A summary of the risk of bias across the trials is presented in Additional file 5: Figure S2 and an overview of the risk of bias per RCTs is presented in Additional file 6: Figure S3. Given the limited variation in the blinding of patients across the RCTs, it was determined that there was insufficient data to assess this assumption in terms of a meta-regression. Similarly, since so many of the RCTs were open-label, a sensitivity analysis to exclude these studies was deemed not to be feasible. Therefore, in order to synthesize the trials in the ‘All Evidence’ base case network, it would be necessary to assume that differences in the study design or quality did not act as a treatment effect modifier, which may bias the treatment estimates.

**Figure 5:**
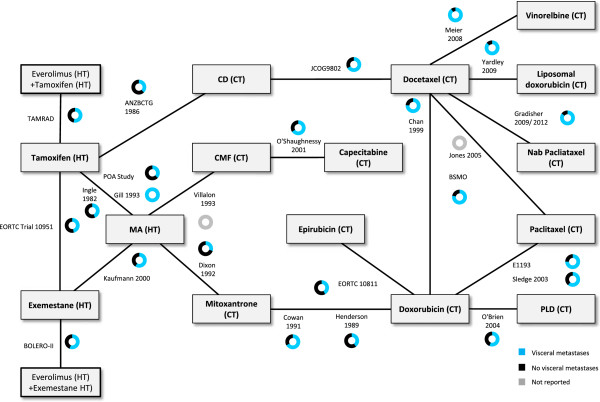
**Network of included RCTs for the base case PFS based on Kaplan Meier curves: visceral metastases.** CD, cyclophosphamide + doxorubicin; CMF, cyclophosphamide + methotrexate + 5-fluorouracil; CT, chemotherapy; HT, hormonal therapy; MA, megestrol acetate; PFS, progression-free survival; PLD, pegylated liposomal doxorubicin; RCT, randomized controlled trial*.*

Differences were identified in terms of the distribution of patient characteristics within and across the treatment comparisons with respect to the potential treatment effect modifiers for ER status (Figure 4), exposure to prior hormonal therapies [see Additional file 7: Figure S4], exposure to prior chemotherapies [see Additional file 8: Figure S5] and visceral metastases (Figure 5). Although a meta-regression to adjust for each of these differences in a separate analysis may be possible, since there were such a large number of studies where the distribution of patient characteristics was not reported, it is likely that these adjustments will be inadequate. Sensitivity analyses to exclude specific studies were not planned given the substantial variation in the differences across the treatment comparisons and the reliance of the network on several older studies comparing hormonal therapy to chemotherapy that did not report several patient characteristics. Therefore, there is a risk that treatment estimates based on an NMA of the base case network will be biased, as the results would only be valid if it can be assumed that differences in the distribution of patient characteristics do not act as treatment effect modifiers (that is, ER status, exposure to prior hormonal therapies, exposure to prior chemotherapies and visceral metastases). Differences in terms of performance status and age were considered less prominent and are summarized in Table 1. Given the variation observed, sensitivity analyses to exclude outlier studies were planned.

**Table 1:** Overview of variation in potential treatment effect modifiers in the RCTs included in the progression-free survival analysis

**Category**	**Study**	**Comparison**	**N**	**ER+**	**ER NA or ER unknown**	**Failed HT adjuvant/ metastatic**	**Failed CT advanced/ metastatic**	**Visceral meta-stases**	**ECOG 0 or 1**	**Median age (years)**	**Post-meno-pausal**	**HER2-**	**Type prior HT**
**Comparisons**	Baselga 2012, BOLERO-II [24]	EXE vs. EVE + EXE	724	100%	0%	100%	NR	56%	96%	62	100%	100%	AI^a^
	Bachelot 2012, [28] TAMRAD	TAM vs. EVE + TAM	111	100%	NR	100%	24%	48%	92%	63	100%	95%	AI^a^
HT vs. HT	Paridaens 2008, [60-62] EORTC-10951	EXE vs. TAM	371	89%	5%	22%	33%	47%	87%	62	100%	NR	TAM
HT vs. HT	Kaufmann 2000 [51-53]	EXE vs. MA	769	68%	32%	100%	17%	59%	NR	65	100%	NR	TAM
HT vs. HT	Muss 1985, [55,56] POASTUDY	TAM vs. MA	136	58%	34%	3%	10%	37%	79%	62	100%	NR	NR
HT vs. HT	Ingle 1982 [48]	TAM vs. MA	55	NR	NR	0%	NR	44%	79%	49	48%	NR	NA
HT vs. HT	Gill 1993 [44]	TAM vs. MA	118	40%	45%	0%	0%	53%	NR	NR	100%	NR	NA
HT vs. CT	ANZBCTG 1986 [40]	TAM vs. CD	226	16%	73%	NR	NR	38%	60%	NR	100%	NR	NR
HT vs. CT	Dixon 1992 [43]	MA vs. MZ	60	20%	10%	100%	0%	30%	100%	61	100%	NR	TAM
HT vs. CT	Villalon 1993 [64]	MA vs. CMF	48	NR	NR	NR	0%	NR	NR	NR	83%	NR	NR
CT vs. CT	Cowan 1991 [68]	MZ vs. DOX	237	40%	13%	55%	59%	67%	73%	NR	83%	NR	NR
CT vs. CT	Henderson 1989 [47]	MZ vs. DOX	325	30%	20%	52%	63%	40%	71%	NR	85%	NR	NR
CT vs. CT	Katsumata 2009, [50] JCOG9802	CD vs. DOC	293	35%	8%	100%	0%	67%	95%	54	NR	NR	NR
CT vs. CT	O'Shaughenessy 2001 [58]	CMF vs. CAP	93	NR	NR	91%	0%	66%	NR	70	100%	NR	49% TAM
CT vs. CT	Chan 1999 [65]	DOX vs. DOC	326	NR	NR	71%	58%	76%	NR	NR	NR	NR	NR
CT vs. CT	Paridaens 2000 [59]	DOX vs. PAC	331	24%	37%	74%	0%	75%	91%	55	NR	NR	NR
CT vs. CT	Sledge 2003, [63] E1193	DOX vs. PAC	453	46%	29%	60%	0%	61%	85%	58	NR	NR	NR
CT vs. CT	Bontenbal 1998, [42] EORTC 10811	DOX vs. EPI	232	NR	NR	36%	98%	42%	73%	56	92%	NR	NR
CT vs. CT	O'Brien 2004 [57]	DOX vs. PLD	509	40%	37%	NR	0%	56%	89%	58	62%	NR	NR
CT vs. CT	Jones 2005 [49]	DOC vs. PAC	449	56%	44%	60%	58%	NR	NR	56	88%	NR	NR
CT vs. CT	Beuselinck 2010, [41] BSMO	DOC vs. PAC	70	42%	NR	NR	81%	78%	84%	NR	100%	NR	NR
CT vs. CT	Gradishar 2009 [45,46]	DOC vs. Nab-PAC	148	NR	NR	NR	0%	91%	97%	NR	81%	NR	NR
CT vs. CT	Yardley 2009 [66]	DOC vs. L-DOX	102	63%	0%	60%	0%	87%	91%	63	NR	NR	NR
CT vs. CT	Meier 2008 [54]	DOC vs. VIN	120	60%	NR	NR	90%	90%	74%	60	NR	NR	NR

^a^AI, aromatase inhibitors included letrozole and anastrozole. CD, cyclophosphamide + doxorubicin; CMF, cyclophosphamide + methotrexate + fluorouracil; CAP, capecitabine; CT, chemotherapy; DOC, docetaxel; DOX, doxorubicin; ECOG, European Co-operative Oncology Group; ER, estrogen receptor; EPI, epirubicin; EVE, everolimus; EXE, exemestane; HER2, human epidermal growth factor receptor type 2; HT, hormonal therapy; L-DOX, liposomal doxorubicin; MA, megestrol acetate; MZ, mitrozantrone; PAC, paclitaxel; PLD, pegylated liposomal doxorubicin; TAM, tamoxifen; VIN, vinorelbine*.*

Beyond the differences identified based on the pre-defined potential treatment effect modifiers, differences were also detected in terms of post-menopausal status, HER2 status, and types of prior hormonal therapies, which are also summarized in Table 1. Sensitivity analyses were planned to exclude studies that were outliers in terms of post-menopausal status [48]. However, no sensitivity analyses were possible in terms of HER2 status, as only the everolimus trials reported this characteristic. Similarly, sensitivity analyses were not planned for differences identified in terms of the type of prior hormonal therapy given the limited number of studies that reported this information: patients in the everolimus trials received prior aromatase inhibitor (letrozole or anastrozole), whereas patients in other studies received an estrogen receptor antagonist (TAM) or this information was not reported.

#### Part C - Differences in baseline risk that are associated with the relative treatment effect

The RCTs in the network were conducted between 1978 and 2012. Therefore, there may be a risk that differences attributable to changes in clinical practice over time could influence the treatment effect, which may justify adjusting for baseline risk. However, in this network there was no clear placebo or standard of care to provide a common treatment arm in order to assess baseline risk. TAM was selected as the baseline comparator of the analysis given the proximity to everolimus and the larger number of RCTs compared to TAM in the network. Additional file 9: Figure S6 presents the individual study results for PFS as extracted from the Kaplan Meier curves per treatment for the included RCTs. This illustration suggests that there is some variation in the baseline risk for TAM, although it is unclear whether these differences are associated with baseline risk. Since there was no clear reference treatment that was common to all (or most) comparisons in the network, adjusting for differences in baseline risk in a meta-regression was not considered. Furthermore, no clear outliers were identified based on the observed variation in baseline risk. Consequently, an NMA combining the RCTs in the base case is at risk of bias due to differences in clinical practice over time, although the role of baseline risk on the treatment effect is not clear.

#### Part D - Differences in observed treatment effects

Additional file 10: Figure S7 presents the PFS results by study. In the network of evidence there are only four comparisons that are supported by multiple RCTs. For the three RCTs comparing MA versus TAM [44,48,56] the differences consistently favor TAM although there is some variation in the magnitude of the observed effect. For the comparison of doxorubicin versus MZ, both RCTs suggest that there is very little difference in PFS between the treatments [47,68], with doxorubicin showing a slightly more favorable result. However, in the comparison of paclitaxel versus doxorubicin the study by Paridaens *et al*. [59,60] favors doxorubicin over paclitaxel, whereas the study by Sledge *et al*. [63] suggests the opposite, although differences in both cases are minimal. Similarly, for the comparison of paclitaxel versus docetaxel the study by Jones *et al*. [49] suggests docetaxel is favored over paclitaxel, whereas the study by Beuselinck *et al*. [41] found very little difference between the treatments and the curves cross each other more than once. Therefore, within the pairwise comparison some heterogeneity was identified, primarily with respect to the chemotherapy comparisons, although the differences were not substantial. The between-study standard deviation resulting from the analysis was 0.41 (95% credible interval (CrI): 0.22, 0.81), which suggests significant between study heterogeneity was present that was confirmed by the improved model fit of the random effects model over the fixed effects model based on the DIC (FE = 3729.0; RE = 3684.0). These results confirm the presence of heterogeneity in the network.

Given that there are three ‘closed loops’ within the network, an unrelated means (UM) analysis [4] was planned to assess whether results for each pairwise comparison were consistent with the estimates from the NMA which included both the direct and indirect evidence. However, due to the limited amount of data in the network, it was expected that there would be insufficient power to detect a significant difference between the NMA and UM models.

#### Results of the NMA

**Figure 6:**
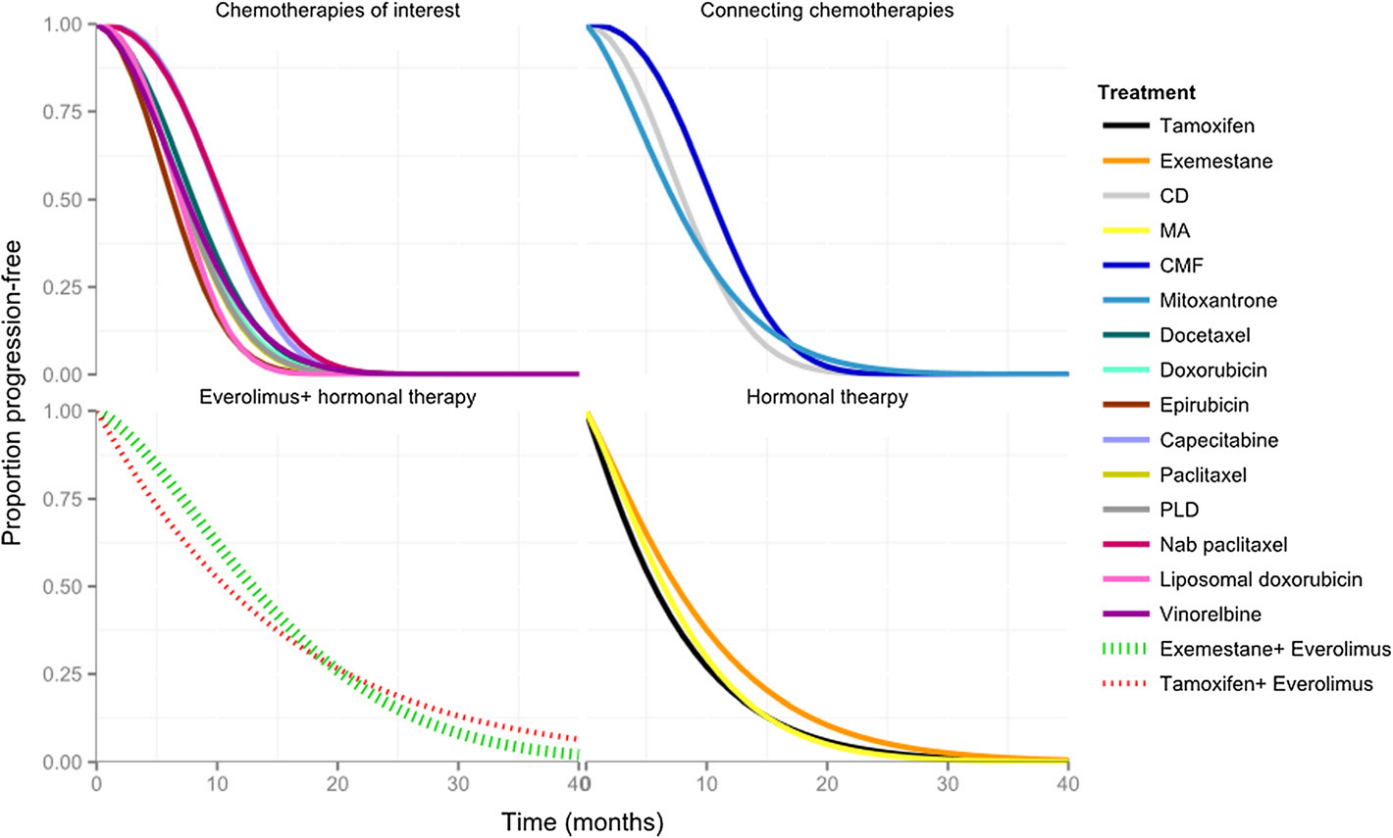
**All Evidence network: PFS over time for each group of treatments as obtained with random effects Weibull network meta-analysis model with time-varying hazard ratios (up to 40 months).** CD, cyclophosphamide + doxorubicin; CMF, cyclophosphamide + methotrexate + 5-fluorouracil; CT, chemotherapy; HT, hormonal therapy; MA, megestrol acetate; PFS, progression-free survival; PLD, pegylated liposomal doxorubicin.

Results are presented in terms of PFS curves over time for each intervention (Figure 6) and the PFS hazard ratios (over time) for each intervention relative to TAM [see Additional file 11: Figure S8]. Based on the PFS survival functions, the mean PFS was estimated at 20 and 40 months, respectively (Table 2). The mean PFS reflects the area under the PFS survival curve to the left of each time point up until the corresponding follow-up time point of interest. This represents a summary measure of PFS that does not require the curves to be fully extrapolated (that is, up until all patients have progressed). Additional detail regarding the interpretation of survival outcomes in the context of a NMA based on fractional polynomial models is discussed by Cope *et al*. [69]. All figures summarizing the results are presented up until 40 months, at which point a majority of the patients are expected to have progressed. Given the large number of curves, the treatments are grouped depending on the comparison: chemotherapies of interest, the connecting chemotherapies (that is, RCTs that connect hormonal therapies to chemotherapies), everolimus in combination with hormonal therapy, and hormonal therapy.

**Table 2:** All evidence network: mean PFS per intervention and difference in expected PFS for everolimus versus alternatives

**Treatment**	Mean PFS at 20 months*	**95% CrI**	Mean PFS at 40 months*	**95% CrI**
Tamoxifen	6.71	(5.31; 8.29)	7.05	(5.48; 9.11)
Exemestane	8.36	(4.55; 12.63)	9.04	(4.60; 16.47)
CD	7.97	(5.00; 11.91)	7.98	(5.00; 12.50)
MA	7.18	(4.51; 10.38)	7.42	(4.54; 11.85)
CMF	10.14	(5.85; 15.76)	10.23	(5.85; 20.36)
Mitoxantrone	7.71	(3.43; 13.03)	7.91	(3.43; 16.01)
Docetaxel	7.92	(4.37; 12.45)	7.93	(4.37; 13.30)
Doxorubicin	7.35	(3.96; 12.09)	7.36	(3.96; 12.80)
Epirubicin	6.16	(2.53; 12.17)	6.16	(2.53; 12.96)
Capecitabine	9.90	(5.03; 16.67)	9.96	(5.04; 22.43)
Paclitaxel	7.10	(3.78; 11.90)	7.11	(3.78; 12.50)
PLD	7.14	(3.09; 13.56)	7.15	(3.09; 15.12)
Nab paclitaxel	10.10	(5.42; 15.75)	10.15	(5.42; 18.33)
Liposomal doxorubicin	6.66	(3.12; 12.00)	6.66	(3.12; 12.38)
Vinorelbine	7.55	(2.99; 14.28)	7.60	(2.99; 17.71)
Exemestane + Everolimus	12.21	(6.21; 16.98)	14.14	(6.25; 26.72)
Tamoxifen + Everolimus	10.85	(5.16; 15.59)	13.62	(5.36; 24.79)

*As obtained with random effects Weibull network meta-analysis model with time-varying hazard ratios and no covariates.

CD, cyclophosphamide + doxorubicin; CMF, cyclophosphamide + methotrexate + fluorouracil; MA, megestrol acetate; PFS, progression-free survival; PLD, pegylated liposomal doxorubicin; 95% CrI, 95% credible interval.

Results suggest that the hazard ratios for vinorelbine, CD, docetaxel, doxorubicin, PLD, paclitaxel, nab-paclitaxel, CMF, epirubicin, capecitabine and liposomal doxorubicin increased over time versus TAM, whereas the hazard ratios for everolimus + TAM, everolimus + EXE, EXE, MA and MZ were relatively constant over time versus TAM. Hence, a proportional hazard assumption is not valid, and, as such, results based on a constant hazard ratio model should be interpreted with caution. Based on the NMA using Weibull time-varying hazard ratio model, everolimus in combination with either EXE or TAM is expected to be at least as good as the alternative chemotherapies of interest. However, given the differences identified in terms of treatment doses, outcome definitions and potential treatment effect modifiers, there is a risk that the indirect treatment estimates are potentially biased. Adjusting for baseline risk did not provide a useful approach for the network of RCTs given the small number of studies including TAM, and identifying potential inconsistencies in the network was challenging given the low power and the number of comparisons included in the closed loops.

## Discussion

The aim of this study was to propose a more structured process to assess the feasibility of performing a valid NMA. The suggested procedure builds on existing recommendations for NMAs and provides more explicit guidance regarding the questions that should be answered at each step. The process is designed to be stepwise, with the initial stages focused on the clinical differences (that is, related to treatments, outcomes, study design and patients) and the later stages focused on evaluating the observed outcomes. Parts A and B involve an assessment of the clinical heterogeneity in terms of treatment, outcome, study and patient characteristics. Parts C and D involve an evaluation of the differences within and across the direct pairwise comparisons in terms of baseline risk and observed treatment effects. This means that it may be decided that an NMA is not feasible after the initial stage, without having assessed heterogeneity or inconsistency. If the decision is made to complete the full feasibility assessment, the available data should be illustrated and the underlying assumptions should be clearly stated, thereby improving the transparency and facilitating an interaction between methodologists and clinicians. While this process does not avoid the need for subjective decisions, it allows decision-makers or researchers to critically analyze each choice as well as to update an analysis using a different approach without necessarily having to repeat the entire process.

The final step of any NMA is to critically assess the findings. Recently guidance to facilitate this process has been developed, including the International Society for Pharmacoeconomics and Outcome Research ‘instrument to assess the relevance and credibility of a NMA’ [70], a ‘reviewer’s checklist’ for evidence synthesis for treatment efficacy used in decision-making [71], as well guidance on ‘how to use an article reporting (Grading of Recommendations and Evaluation (GRADE)) a multiple treatment comparison meta-analysis’ [72]. Additionally, the GRADE process to assess meta-analyses has recently been updated by Cochrane to address the NMA more specifically. The current process for assessing the feasibility does not provide explicit guidance regarding the types of tools to be used for this process, but there seems to be a shared focus on some key principles that should be assessed, including the magnitude of the treatment effects, the uncertainty in the estimates and the risk of bias due to the quality of the RCTs as well as any differences in the distribution of treatment effect modifiers across direct treatment comparisons.

In the case study comparing everolimus to alternative chemotherapies in terms of PFS for women with ABC the feasibility of the NMA was determined to be limited. Although it was possible to achieve a connected network of RCTs for the comparisons of interest, differences were identified in terms of the treatment doses and the outcome definitions, which could be explored by excluding outlier studies. However, differences were also identified with respect to the pre-defined treatment effect modifiers as well as *post-hoc* differences in specific patient characteristics that were not possible to explore based on the available data. Some variation in baseline risk within trials including TAM was observed, as was some heterogeneity in the treatment effects, whereas the inconsistency was challenging to assess in this network. In conclusion, given the differences identified in potential treatment effect modifiers which cannot be explored, there is a substantial risk that differences in these potential treatment effect modifiers may introduce bias, threatening the overall validity of the NMA, which reflects a limitation of the available data. Despite the limited feasibility of the case study, it was decided to perform the NMA for exploratory purposes. The point estimates from the analysis suggest that everolimus in combination with EXE or TAM is at least as efficacious as the chemotherapies of interest in terms of PFS. However, the comparison of interest is linked through several indirect treatment comparisons, which led to substantial uncertainty in the treatment estimates. We would advise caution regarding the interpretation of the results given the conclusion of the feasibility assessment.

The decision to proceed with the NMA can be criticized in light of the findings from the feasibility assessment. However, there is an immediate need for evidence from decision-makers given the context of the research question, as well as a potential long-term gap in the evidence, which suggests this NMA may provide the best available evidence. For example, findings from the current NMA may provide a more robust result based on the available evidence in comparison to a previous ‘naïve chained indirect analysis’ that multiplied a pooled hazard ratio for chemotherapy versus endocrine therapy (from the meta-analysis by Wilken *et al.*) by a hazard ratio for everolimus in combination with TAM versus TAM (based on the TAMRAD trial and assumed to be the same as everolimus in combination with EXE to EXE) [73]. Although there is a risk that results of the NMA will be over-interpreted, we would argue that the purpose of the feasibility assessment is to ensure that the underlying assumptions and limitations of the NMA are clearly communicated. Further, NMA results may help to quantify the between-study variability (and possibly the inconsistencies in the evidence base in some cases), thereby providing a more complete exploration of heterogeneity, which may generate further hypotheses [7]. Finally, in some cases, results of an NMA may actually help to trigger a response from clinical experts regarding the plausibility of the underlying assumptions, which may otherwise be more difficult to reveal. In general, we would advise consideration regarding the value of exploratory analyses against the risk of over-interpretation.

The case study of everolimus for women with ABC provides a unique opportunity to illustrate the challenges associated with evaluating the feasibility of a NMA given that this new treatment reflects a step-change in clinical practice. In such cases where a new treatment introduces an additional step in the traditional treatment pathway, it may be necessary to compare the current treatment pathway (in the absence of the new treatment) with the anticipated treatment pathway (including the new treatment). When there are no trials available comparing the current treatment pathway to the anticipated treatment pathway, this often implies a comparison between the new treatment and the usual treatment used as the ‘next step’ in the treatment pathway. However, by definition, a new treatment that delays the next step in a treatment pathway is designed to target a less severe population. Consequently, there is an inherent risk that the patient characteristics of the RCTs available for the new treatment are not comparable to those patients in the RCTs evaluating the ‘next step’. Additionally, as new treatments become more targeted based on genetic differences in receptors, it may be difficult to compare new trials evaluating a subset of patients with older trials including a full population (that may not report the receptors of interest). Despite these limitations, it may be decided to combine the direct and indirect results and to perform an NMA given the absence of evidence regarding the comparison of interest and the need for clinicians and health technology bodies such as NICE to make decisions. The tendency to perform an NMA in the context reinforces the importance of the feasibility assessment process. Moreover, this case study identifies a clear need for a new trial comparing the everolimus to chemotherapy, or a comparison of the alternative treatment pathways with and without everolimus (that is, everolimus followed by chemotherapy versus placebo followed by chemotherapy).

One of the main limitations of the case study is that overall survival was not assessed. The current study focused on PFS given the available data for everolimus at the time of the feasibility assessment. In comparison to overall survival, PFS is not susceptible to confounding by differences in subsequent treatments across the studies, although there is a risk of assessment bias with PFS. Therefore, overall survival, as well as the safety and adverse events of these agents, should be considered in addition to the results of the current NMA. Another limitation is that the current case study was based on a research question focused on the comparison of everolimus versus chemotherapy. However, the original scope of the research question as defined by NICE also included fulvestrant as a comparator of interest. A separate NMA has been performed by Bachelot *et al*. in order to address this comparison of interest among women with ER+ ABC following progression or recurrence after endocrine therapy [74]. Although ideally all of the comparisons of interest should be included in one simultaneous analysis, there is a clear justification for a separate analysis in this case given the challenge of comparing everolimus to chemotherapy.

It should be noted that this feasibility process has some limitations. In the initial stages (parts A and B) it may not be necessary to extract the outcomes of interest from all studies, thereby improving the efficiency of the process. However, it is necessary to assess whether there is a sufficient amount of information reported regarding the outcome and its measure of uncertainty, which requires decision rules regarding the calculation of treatment differences or the estimates of uncertainty that may be particularly challenging to define *a priori* for continuous endpoints depending on the available information. Similarly, if imputation will be used to assess uncertainty measures, a threshold regarding the amount of missing information that will be permitted may be necessary. However, pre-specifying decision-rules for all possible types of endpoints, including optimal thresholds for the amount of data required for covariate analyses may be challenging. Although some research has evaluated alternative imputation methods for NMAs [75], to our knowledge alternative thresholds for missing data depending on the type of outcome requires further research.

Although the current case study was based on a complex network structure, in ‘star’ shaped networks, involving several trials with a common comparator (such as placebo), we would emphasize the importance of assessing whether differences in baseline risk exist and can be adjusted (part C). A plot of the difference measure versus the baseline risk is useful to help illustrate the variation in the baseline risk, as well as the relationship between the difference and baseline risk for each treatment. Even in cases where head-to-head trials are included in the network, it is possible to predict a placebo-arm on the basis of the other trials [15].

The current framework suggests a separate process for each outcome (and time point) of interest based on evidence available from RCTs identified from a systematic review regarding a comparative efficacy or safety question. However, undergoing the outlined feasibility process is expected to be very time consuming, and it may be more realistic to assess multiple outcomes in parallel, particularly when they are related to the same endpoints or underlying concepts. The case study explores the feasibility of a NMA based on a synthesis of Kaplan Meier curves; however, this process can be applied to any type of endpoints (that is, binary, continuous or rate outcomes). For binary endpoints it may be important to consider whether differences in follow-up are expected to act as a treatment effect modifier and, if so, to what extent different follow-up (or time points) can be combined. Similarly, for continuous outcomes, the range of time points at follow-up than can be considered comparable should be clearly addressed. It may also be important to consider models that combine multiple time points (repeated measures) or outcomes identified within the systematic review, particularly in cases where the initial feasibility assessment suggests a NMA may not be feasible. For example, in the context of ABC, a multi-state model that accounts for PFS and overall survival (as well as the relationship between the outcomes) may provide more information (and possibly more precision).

Another possible extension of the current process would be to consider a broader evidence base if a network is deemed not to be feasible. Depending on how the research question was defined, it may be important to assess whether additional indirect evidence may be available by broadening the comparators of interest, although this consideration should be offset by the risk of introducing different populations in terms of the distribution of treatment effect modifiers. Similarly, it may be possible to integrate non-randomized evidence using more informative prior distributions [4] or individual patient data from RCTs [5,76-78] or non-randomized studies [79-81], which may influence the feasibility of an analysis. Furthermore, it may be possible to elicit bias distributions when there is insufficient data for a meta-regression where experts provide information regarding internal and external biases in order to adjust the study-specific treatment effect [82] as cited in [20]. However, these methods to combine multiple time points, outcomes and study designs are evolving currently and require further research. Therefore, the current process may provide a useful starting point to identify the need for a more complicated approach.

## Conclusions

In conclusion, the process outlined to assess the feasibility of a NMA provides a stepwise framework that will help to ensure that the underlying assumptions are systematically explored and that the risks (and benefits) of pooling and indirectly comparing treatment effects from RCTs for a particular research question are transparent.

## Abbreviations

ABC: advanced breast cancer; AI: aromatase inhibitor; CD: cyclophosphamide + doxorubicin; CMF: cyclophosphamide + methotrexate + fluorouracil; CT: chemotherapy; DIC: deviance information criterion; ECOG: European Co-operative Oncology Group; ER+: estrogen receptor positive; EVE: everolimus; EXE: exemestane; HER2-: human epidermal growth factor receptor 2 negative; HR-status: hormone receptor status; HT: hormonal therapy; MA: megestrol acetate; MZ: mitrozantrone (mitoxantrone); NICE: National Institute for Health and Clinical Excellence; NMA: network meta-analysis; NSAI: non-steroidal aromatase inhibitors; PFS: progression-free survival; PLD: pegylated liposomal doxorubicin; RCT: randomized controlled trial; TAM: tamoxifen; TTF: time to failure; TTP: time to progression; UM: unrelated mean; 95% CrI: 95% credible interval.

## Competing interests

SC, and BS are employees of Mapi and received funding from Novartis for the study. JJ is a former employee of Mapi and received funding from Novartis for the study. JZ and SS are full time employees of Novartis Pharmaceutical Corp and have shares in the company. PS received no compensation for working on this manuscript. PS has no competing interests.

## Authors’ contributions

All authors participated in the development of this manuscript. SC, JJ and BS participated in all stages of the study design, systematic literature view, statistical analyses and manuscript development. JZ, SS, and PS participated in the study design and coordination and helped to draft the manuscript. All authors read and approved the final manuscript.

## Pre-publication history

The pre-publication history for this paper can be accessed here:

http://www.biomedcentral.com/1741-7015/12/93/prepub

## Supplementary Material

Additional file 1 Search Strategy. Click here for file

Additional file 2: Table S1 Trial names, publications and interventions assessed. Click here for file

Additional file 3: Figure S1 Network of included RCTs for the base case progression-free survival analysis based on Kaplan Meier curves. Click here for file

Additional file 4: Table S2 Treatment doses and schedules for the RCTs included in the network. Click here for file

Additional file 5: Figure S2 Network of included RCTs for the base case PFS based on Kaplan Meier curves: risk of bias summary. Click here for file

Additional file 6: Figure S3 Network of included RCTs for the base case PFS based on Kaplan Meier curves: risk of bias per trial. Click here for file

Additional file 7: Figure S4 Network of included RCTs for the base case PFS based on Kaplan Meier curves: prior hormonal therapy. Click here for file

Additional file 8: Figure S5 Network of included RCTs for the base case PFS based on Kaplan Meier curves: prior chemotherapy. Click here for file

Additional file 9: Figure S6 PFS as extracted from Kaplan Meier curves for individual randomized controlled trials included by treatment. Click here for file

Additional file 10: Figure S7 PFS as extracted from Kaplan Meier curves by individual randomized controlled trials included by study. Click here for file

Additional file 11: Figure S8 All Evidence network: progression-free survival hazard ratio over time for each treatment group relative to tamoxifen as obtained with random effects Weibull network meta-analysis model with time varying hazard ratios and no covariates (up to 40 months). Click here for file
