# Supplementary material for: A process for assessing the feasibility of a network meta-analysis: a case study of everolimus in combination with hormonal therapy versus chemotherapy for advanced breast cancer
Source: BMC Med. 2014 Jun 5;12:93. doi: 10.1186/1741-7015-12-93 (PMC4077675; doi:10.1186/1741-7015-12-93)
Supplement: Additional file 1 — Search Strategy. [file 1741-7015-12-93-S1.pdf]

Search strategy

*Search performed***:** March 1, 2013

*Databases***:** Embase <1988 to 2013 Week 08>, Ovid MEDLINE(R) In-Process & Other Non-Indexed Citations and Ovid MEDLINE(R) <1946 to Present>

*Search terms***:**

1	"randomized controlled trial".pt. (341377)
2	(random$ or placebo$ or single blind$ or double blind$ or triple blind$).ti,ab.
3	(retraction of publication or retracted publication).pt.
4	1 or 2 or 3
5	(animals not humans).sh.
6	((comment or editorial or meta-analysis or practice-guideline or review or letter or journal correspondence) not "randomized controlled trial").pt.
7	(random sampl$ or random digit$ or random effect$ or random survey or random regression).ti,ab. not "randomized controlled trial".pt.
8	5 or 6 or 7
9	4 not 8
10	(random$ or placebo$ or single blind$ or double blind$ or triple blind$).ti,ab.
11	RETRACTED ARTICLE/
12	10 or 11
13	(animal$ not human$).sh,hw.
14	(book or conference paper or editorial or letter or review).pt. not exp randomized controlled trial/
15	(random sampl$ or random digit$ or random effect$ or random survey or random regression).ti,ab. not exp randomized controlled trial/
16	13 or 14 or 15
17	12 not 16
18	9 or 17
19	exp breast neoplasms/
20	((breast or mammary or mammarian) and (cancer$ or carcinoma$ or neoplasm$ or tumo?r$ or malignan$)).tw.
21	exp Breast Cancer/
22	19 or 20 or 21

23	(Advanc$ or metasta$3 or stage 3 or stage III or stage 4 or stage IV).ti,ab.
24	22 and 23
25	chemotherap*.mp.
26	exp Drug Therapy/
27	(chemotherap* adj6 alone).mp
28	endocrine therap*.mp
29	chemotherapy/
30	chemotherap*.mp. [mp=ti, ab, sh, hw, tn, ot, dm, mf, dv, kw, nm, kf, ps, rs, ui]
31	(endocrine and therap*).mp. [mp=ti, ab, sh, hw, tn, ot, dm, mf, dv, kw, nm, kf, ps, rs, ui]
32	(megestrol acetate or Megace or tamoxifen or Nolvadex or Istubal or Valodex or exemestane or Aromasin or everolimus or Afinitor or cyclophosphamide or Endoxan or Cytoxan or Neosar or Procytox or Revimmune or methotrexate or fluorouracil or 5-fluorouracil or Efudex or doxorubicin or Adriamycin or mitoxantrone or mitozantrone or Novantrone or epirubicin or Ellence or paclitaxel or Taxol or docetaxel or Taxotere or lipsosomal doxorubicin or Doxil or nab-paclitaxel or Abraxane or eribulin or Halaven or capecitabine or Xeloda).ti,ab.
33	25 or 26 or 27 or 28 or 29 or 30 or 31 or 32
34	18 and 24 and 33
35	limit 34 to english language
36	limit 35 to human
37	limit 36 to humans
38	remove duplicates from 37
