# Supplementary material for: A process for assessing the feasibility of a network meta-analysis: a case study of everolimus in combination with hormonal therapy versus chemotherapy for advanced breast cancer
Source: BMC Med. 2014 Jun 5;12:93. doi: 10.1186/1741-7015-12-93 (PMC4077675; doi:10.1186/1741-7015-12-93)
Supplement: Additional file 2: Table S1 — Trial names, publications and interventions assessed. [file 1741-7015-12-93-S2.pdf]

Supplemental Table 2. Treatment doses and schedules for the RCTs included in the network

Trial	Treatment	Dose and Schedule
O'Shaughnessy 2001	capecitabine	1255mg/m2 2X daily (2 weeks on/1 week off) (orally)
JCOG9802 2009	cyclophosphamide + doxorubicin	500mg/m2 + 40mg/m2 Q3W
ANZBCTG 1986	cyclophosphamide + doxorubicin	750mg/m2 + 50mg/m2 Q3W (IV)
Villalon 1993	CMF	100mg/m2 days 1 + 14 (orally); 40mg/m2 days 1 + 8 (IV); 600mg/m2 days 1 + 8 (IV); Q4W
O'Shaughnessy 2001	CMF	600mg/m2 (IV); 40mg/m2 (IV); 600mg/m2 (IV); Q3W
Meier 2008	docetaxel	35mg/m2 once weekly X6 every 8 weeks (premed with dexamethasone)
BSMO Study 2010	docetaxel	36mg/m2 QW for 8 weeks, then 3wk on/ 1 wk. off or 2wk on/1 wk. off (IV)
Yardley 2009	docetaxel	36mg/m2 days 1, 8 , 15 Q4W (IV) (premed dexamethasone)
JCOG9802 2009	docetaxel	60mg/m2 Q3W (IV) (premed dexamethasone)
Gradishar 2009	docetaxel	100mg/m2 Q3W (premed with oral corticosteroids)
Chan 1999	docetaxel	100mg/m2 Q3W (IV for 1 hr.) (premed with dexamethasone)
Jones 2005	docetaxel	100mg/m2 Q3W (IV) (premed with dexamethasone)
Cowan 1991	doxorubicin	60mg/m2 Q3W (IV for 15min)
O'Brien 2004	doxorubicin	60mg/m2 Q3W (IV)
E1193 2003	doxorubicin	60mg/m2 Q3W (IV)
EORTC 10811 1998	doxorubicin	74mg/m2 Q3W (IV) (3 week cycle)
Chan 1999	doxorubicin	75mg/m2 Q3W (IV for 15-20 mins)
Henderson 1989	doxorubicin	75mg/m2 Q3W (IV)
Paridaens 2000	doxorubicin	75mg/m2 Q3W (IV)
EORTC 10811 1998	epirubicin	90mg/m2 Q3W (IV) (3 week cycle)
BOLERO-II 2012	everolimus + exemestane	10mg daily + 25mg daily (orally)
TAMRAD 2012	everolimus + tamoxifen	10mg daily + 20mg daily (orally)
BOLERO-II 2012	exemestane	25mg daily (orally)
EORTC-10951 2008	exemestane	25mg daily (orally)
Kaufmann 2000	exemestane	25mg daily (orally)
Yardley 2009	liposomal doxorubicin	40mg/m2 Q4W (IV)
Kaufmann 2000	megestrol acetate	40mg 4X daily (orally)
POA study 1985	megestrol acetate	40mg 4X daily (orally)
Ingle 1982	megestrol acetate	150mg/m2 daily (3 divided doses daily) (orally)
Villalon 1993	megestrol acetate	160mg daily (orally)
Gill 1993	megestrol acetate	160mg daily
Dixon 1992	megestrol acetate	160mg 2X daily
Cowan 1991	mitoxantrone	14mg/m2 Q3W (IV for 10min)

Trial	Treatment	Dose and Schedule
Henderson 1989	mitoxantrone	14mg/m2 Q3W (IV)
Dixon 1992	mitoxantrone	14mg/m2 Q3W (IV) (4 courses)
BSMO Study 2010	paclitaxel	80mg/m2 QW for 8 weeks, then 3wk on/ 1 wk. off or 2wk on/1 wk. off (IV)
Jones 2005	paclitaxel	175mg/m2 Q3W (IV)
E1193 2003	paclitaxel	175mg/m2 Q3W
Paridaens 2000	paclitaxel	200mg/m2 Q3W (IV)
O'Brien 2004	pegylated liposomal doxorubicin	50mg/m2 Q4W (IV)
Ingle 1982	tamoxifen	10mg 2X daily (orally)
POA study 1985	tamoxifen	10mg 2X daily (orally)
Taylor 1986	tamoxifen	10mg 2X daily (orally) (4 week cycle)
TAMRAD 2012	tamoxifen	20mg daily (orally)
EORTC-10951 2008	tamoxifen	20mg daily (orally)
ANZBCTG 1986	tamoxifen	20mg 2X daily (orally)
Gill 1993	tamoxifen	20mg 2X daily (40mg total)
Meier 2008	vinorelbine	30mg/m2 once weekly X 6 every 8 weeks

CD-cyclophosphamide + doxorubicin; CMF- cyclophosphamide + methotrexate + 5-fluorouracil; IV-intravenous; MA- megestrol acetate; mins-minutes; PLD-pegylated liposomal doxorubicin; QW-weekly; Q3W-every three weeks; Q4W-every four weeks; wk-week
