# Supplementary material for: A process for assessing the feasibility of a network meta-analysis: a case study of everolimus in combination with hormonal therapy versus chemotherapy for advanced breast cancer
Source: BMC Med. 2014 Jun 5;12:93. doi: 10.1186/1741-7015-12-93 (PMC4077675; doi:10.1186/1741-7015-12-93)

Supplemental Figure 2. Network of included RCTs for the base case PFS based on Kaplan Meier curves: Risk of bias summary

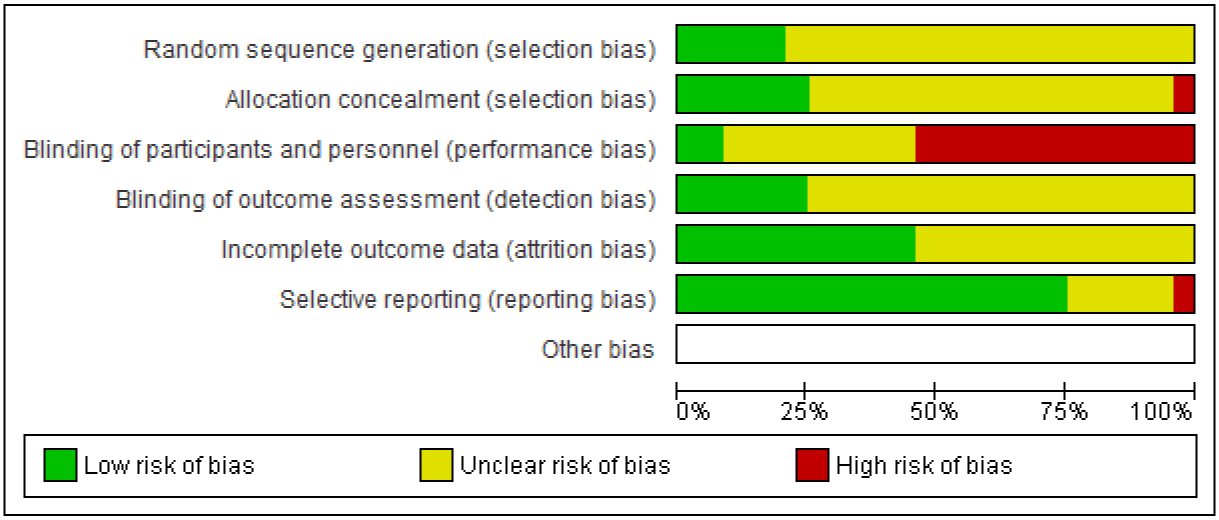

Supplement: Additional file 3: Figure S1 — Network of included RCTs for the base case progression-free survival analysis based on Kaplan Meier curves. [file 1741-7015-12-93-S3.pdf]
