# Supplementary material for: A process for assessing the feasibility of a network meta-analysis: a case study of everolimus in combination with hormonal therapy versus chemotherapy for advanced breast cancer
Source: BMC Med. 2014 Jun 5;12:93. doi: 10.1186/1741-7015-12-93 (PMC4077675; doi:10.1186/1741-7015-12-93)
Supplement: Additional file 4: Table S2 — Treatment doses and schedules for the RCTs included in the network. [file 1741-7015-12-93-S4.pdf]

Supplemental Figure 3. Network of included RCTs for the base case PFS based on Kaplan Meier curves: Risk of bias per trial

	Random sequence generation (selection bias)	Allocation concealment (selection bias)	Blinding of participants and personnel (performance bias)	Blinding of outcome assessment (detection bias)	Incomplete outcome data (attrition bias)	Selective reporting (reporting bias)
ANZBCTG 1986	?	+	−	?	+	+
BOLERO-II	?	?	+	+	?	+
BSMO STUDY	?	?	−	?	?	?
CHAN 1999	?	−	−	?	+	+
COWAN 1991	?	?	?	+	+	+
DIXON 1992	?	?	−	?	?	+
E1193	?	?	?	?	?	+
EORTC-10811	+	?	?	?	?	?
EORTC-10951	+	+	−	?	+	+
GILL 1993	?	+	?	?	?	?
GRADISHAR 2009	?	?	−	+	?	+
HENDERSON 1989	+	+	?	?	+	+
INGLE 1982	?	?	?	?	?	+
JCOG9802	+	+	−	?	+	+
JONES 2005	?	?	−	?	+	+
KAUFMANN 2000	+	+	+	+	?	?
MEIER 2008	?	?	?	?	?	?
O'BRIEN 2004	?	?	−	?	+	+
O'SHAUGHNESSY 2001	?	?	−	+	+	+
PARIDAENS 2000	?	?	−	+	+	+
POA STUDY	?	?	?	?	?	+
TAMRAD	?	?	−	?	?	+
VILLALON 1993	?	?	−	?	?	−
YARDLEY 2009	?	?	?	?	+	+

Red=High risk of bias; Yellow=Unclear risk of bias; Green=Low risk of bias.
