# Supplementary material for: A process for assessing the feasibility of a network meta-analysis: a case study of everolimus in combination with hormonal therapy versus chemotherapy for advanced breast cancer
Source: BMC Med. 2014 Jun 5;12:93. doi: 10.1186/1741-7015-12-93 (PMC4077675; doi:10.1186/1741-7015-12-93)
Supplement: Additional file 5: Figure S2 — Network of included RCTs for the base case PFS based on Kaplan Meier curves: risk of bias summary. [file 1741-7015-12-93-S5.pdf]

Supplemental Figure 4. Network of included RCTs for the base case PFS based on Kaplan Meier curves: Prior hormonal therapy

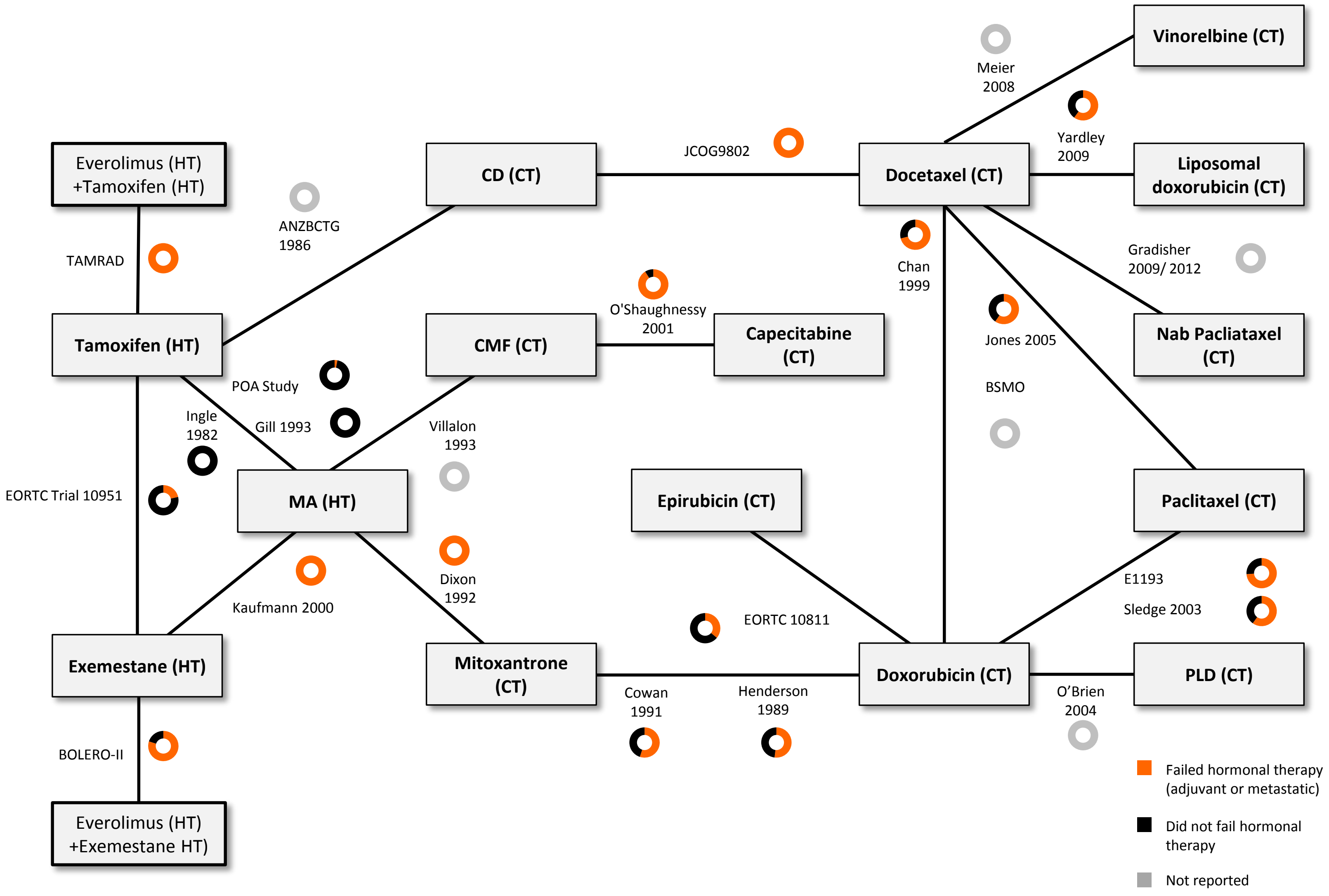

CD- Cyclophosphamide + doxorubicin; CMF- Cyclophosphamide + methotrexate + 5-fluorouracil; CT- Chemotherapy; ER- Estrogen receptor; HT- Hormonal therapy; PLD- Pegylated liposomal doxorubicin
