# Supplementary material for: A process for assessing the feasibility of a network meta-analysis: a case study of everolimus in combination with hormonal therapy versus chemotherapy for advanced breast cancer
Source: BMC Med. 2014 Jun 5;12:93. doi: 10.1186/1741-7015-12-93 (PMC4077675; doi:10.1186/1741-7015-12-93)

Supplemental Figure 6. PFS as extracted from Kaplan Meier curves for individual randomized controlled trials included by treatment

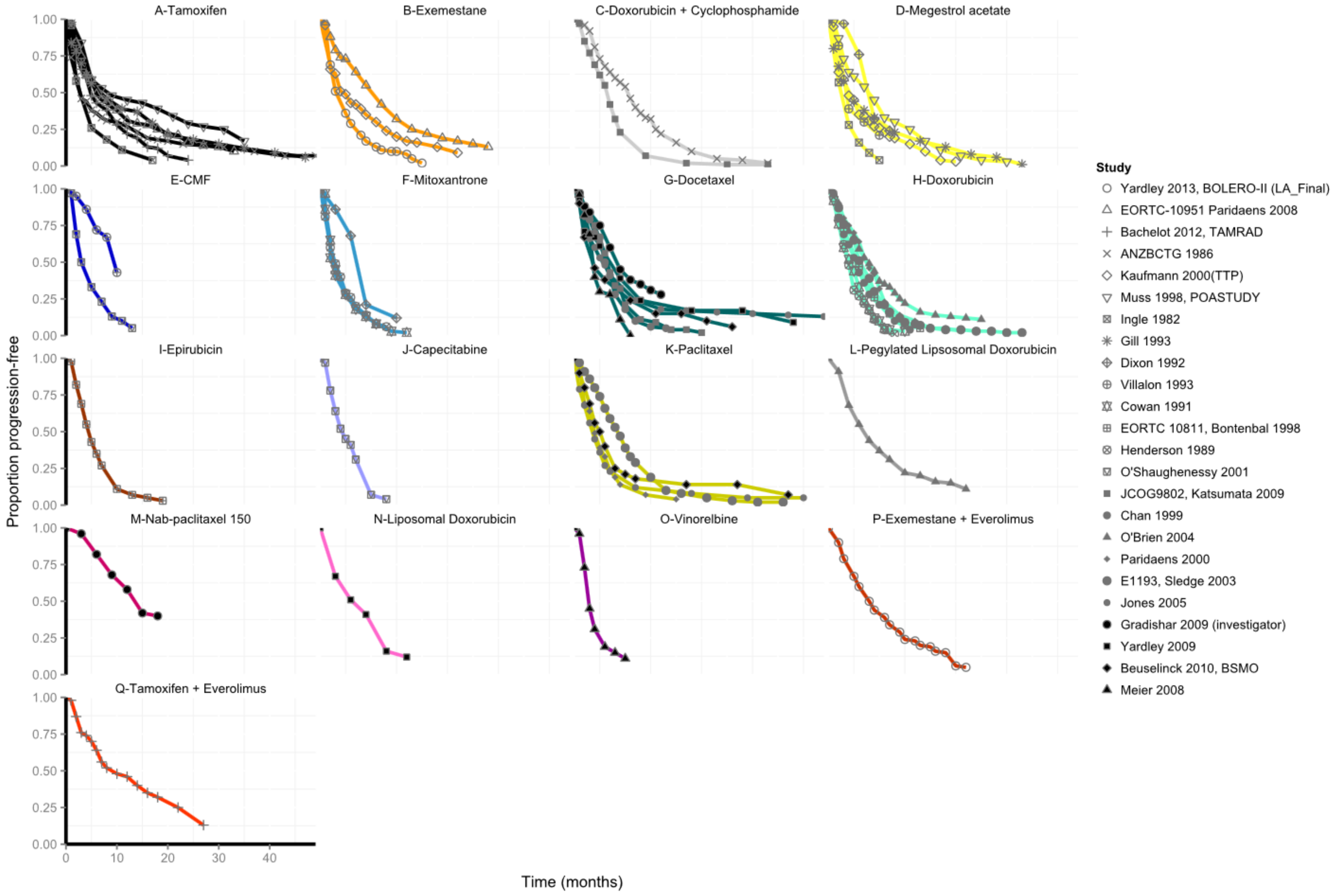

Supplement: Additional file 7: Figure S4 — Network of included RCTs for the base case PFS based on Kaplan Meier curves: prior hormonal therapy. [file 1741-7015-12-93-S7.pdf]
