# Supplementary material for: A process for assessing the feasibility of a network meta-analysis: a case study of everolimus in combination with hormonal therapy versus chemotherapy for advanced breast cancer
Source: BMC Med. 2014 Jun 5;12:93. doi: 10.1186/1741-7015-12-93 (PMC4077675; doi:10.1186/1741-7015-12-93)

Supplemental Figure 8. All Evidence network: Progression-free survival hazard ratio over time for each treatment group relative to tamoxifen as obtained with random effects Weibull network meta-analysis model with time varying hazard ratios and no covariates (up to 40 months)

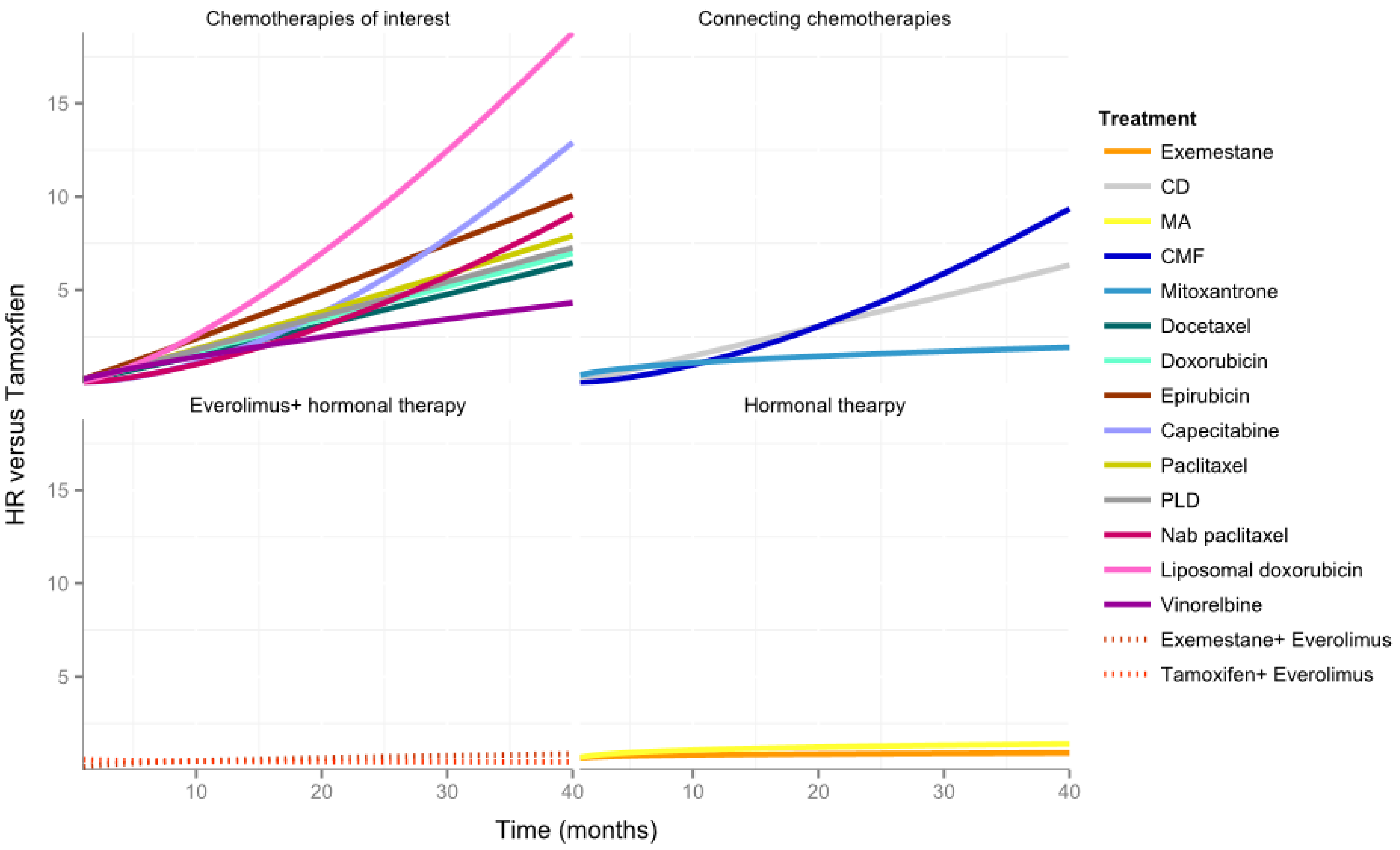

Supplement: Additional file 9: Figure S6 — PFS as extracted from Kaplan Meier curves for individual randomized controlled trials included by treatment. [file 1741-7015-12-93-S9.pdf]
